# Fungal Microbiota in Chronic Airway Inflammatory Disease and Emerging Relationships with the Host Immune Response

**DOI:** 10.3389/fmicb.2017.02477

**Published:** 2017-12-12

**Authors:** Irene Zhang, Steven D. Pletcher, Andrew N. Goldberg, Bridget M. Barker, Emily K. Cope

**Affiliations:** ^1^Pathogen and Microbiome Institute, Northern Arizona University, Flagstaff, AZ, United States; ^2^Department of Otolaryngology Head and Neck Surgery, University of California, San Francisco, San Francisco, CA, United States

**Keywords:** airway microbiome, airway fungal microbiome, mycobiome, host-microbiome interactions, bacterial-fungal interactions, biofilm

## Abstract

The respiratory tract is a complex system that is inhabited by niche-specific communities of microbes including bacteria, fungi, and viruses. These complex microbial assemblages are in constant contact with the mucosal immune system and play a critical role in airway health and immune homeostasis. Changes in the composition and diversity of airway microbiota are frequently observed in patients with chronic inflammatory diseases including chronic rhinosinusitis (CRS), cystic fibrosis, allergy, and asthma. While the bacterial microbiome of the upper and lower airways has been the focus of many recent studies, the contribution of fungal microbiota to inflammation is an emerging research interest. Within the context of allergic airway disease, fungal products are important allergens and fungi are potent inducers of inflammation. In addition, murine models have provided experimental evidence that fungal microbiota in peripheral organs, notably the gastrointestinal (GI) tract, influence pulmonary health. In this review, we explore the role of the respiratory and GI microbial communities in chronic airway inflammatory disease development with a specific focus on fungal microbiome interactions with the airway immune system and fungal-bacterial interactions that likely contribute to inflammatory disease. These findings are discussed in the context of clinical and immunological features of fungal-mediated disease in CRS, allergy, and asthmatic patients. While this field is still nascent, emerging evidence suggests that dysbiotic fungal and bacterial microbiota interact to drive or exacerbate chronic airway inflammatory disease.

## Introduction

Microbial communities associated with host mucosal surfaces play essential and diverse roles in biological processes from metabolism to immune regulation and homeostasis. In return, host inflammatory responses can shape the microbial community by supporting the growth of some microbes while inhibiting others (Kumamoto, 2016). Most studies of host-associated microbiota in health and disease have focused on the bacterial microbiota, despite fungal diseases incurring a substantial infectious disease burden (Huffnagle and Noverr, 2013). Because of this, relatively little is known about the importance and function of the fungal microbiota (mycobiota). Recent advances in culture-independent sequencing approaches have helped elucidate the fungal diversity, burden, and functions associated with mucosal surfaces such as the human intestinal tract, oral cavity, skin, and respiratory tract.

In virtually every body habitat studied to date, niche-specific microbial communities have been identified, including the gastrointestinal and respiratory tracts (Human Microbiome Project Consortium, 2012). Fungi live in host niches as commensals and interact with bacteria and the host as members of the healthy microbiome. While fungal microbiota are numerically small, they are diverse, have large genomes, and potentially act as important keystone species in the microbiome (Huffnagle and Noverr, 2013). Studies have shown that disrupting commensal fungi can affect both local and peripheral immune responses and enhance disease states. In addition, members of the mycobiome can switch from commensalism to pathogenicity, often dependent upon co-colonizing microbial taxa (Cohen et al., 1969; Peleg et al., 2010). Recent studies suggest a role for airway microbial communities in maintaining airway health (Abreu et al., 2012). In this perspective, we focus on the potential role of fungal interactions with the host and bacterial co-colonizers chronic rhinosinusitis (CRS) and lower airway comorbidities.

## Respiratory tract physiology

Like the GI tract (Jeraldo et al., 2012), the respiratory tract is composed of distinct environments that vary according to mucosal architecture and immune responses. Reflective of this, the microbial communities that inhabit the respiratory tract exhibit niche specificity; compositionally distinct communities exist between the nares, sinuses, oral cavity, oropharynx and lower airway in healthy individuals (Lemon et al., 2010; Bassis et al., 2015). The sinonasal cavity, nasopharynx, and trachea are lined with a pseudostratified columnar ciliated epithelium and mucus-producing goblet cells. Lung bronchi and bronchioles are covered by stratified columnar ciliated epithelia and few goblet cells. Mucus secretion and immune effector molecules protect the host from insults by trapping and removing inhaled pathogens and irritants, a process orchestrated by ciliary beating (Cohen, 2006). Airway epithelial cells express pattern recognition receptors (PRRs), including toll-like receptors (TLR) and non-TLR PRRs such as Dectin-1, that can recognize and respond to components of microorganisms (Ryu et al., 2013; Liu et al., 2015).

## Chronic inflammatory diseases of the upper and lower airways

Chronic airway inflammatory diseases including asthma, rhinosinusitis, and rhinitis are substantial health problems resulting in significant healthcare expenditures. Chronic rhinosinusitis (CRS) alone affects approximately 15% of the adult population annually; recent estimates suggest that costs associated with CRS exceed $65 billion each year accounting for 5% of the US healthcare budget (Caulley et al., 2015). Several epidemiological studies suggest that concomitant upper and lower airway disease result from shared pathophysiologic mechanisms (Passalacqua et al., 2001; Ciprandi et al., 2012; Licari et al., 2014).

CRS is clinically defined by duration of symptoms and endoscopic or radiographic evidence of sinus inflammation. Multiple subtypes of CRS have been described based on clinical (Akdis et al., 2013; Orlandi et al., 2016), immunological (Tomassen et al., 2016) and microbiological (Cope et al., 2017) heterogeneity. The role of fungi in the pathophysiology of CRS has long been controversial. While some authors argued that fungi drove inflammation in nearly all CRS patients (Ponikau et al., 2000), this finding has been recently disputed (Orlandi and Marple, 2010; Fokkens et al., 2012). Fungi clearly play a central role in several discrete subtypes of CRS, such as allergic fungal sinusitis and development of fungus balls (deShazo et al., 1997). Fungi found in culture-based studies of CRS patients include members of *Aspergillus, Alternaria, Candida, Cladosporium*, and *Penicillium* (Ponikau et al., 1999). Molecular studies have expanded this list to include fastidious fungi *Malassezia, Curvularia, Schizophyllum*, and *Neocosmospora* (Cleland et al., 2014; Gelber et al., 2016; Zhao et al., 2017). While there are few studies on host-fungal interactions in the upper airways, recent research suggests that defects in epithelial genes might compromise immune barrier function and lead to dysfunctional host immune responses to bacterial or fungal colonization (Tieu et al., 2009). Additionally, fungal glycans (e.g., β-glucan) interact with airway epithelial cells via surface receptors and can act as determinants of allergenicity or induce inflammation (Roy and Klein, 2013). In allergic bronchopulmonary aspergillosis (ABPA), structural abnormalities in the airway epithelium allow fungal spores to breach immune defenses and germinate into hyphae (Chaudhary and Marr, 2011). Upon germination, fungal spores secrete proteases which can disrupt epithelial barrier integrity (Chen et al., 2011). Epithelial barrier dysfunction is frequently observed in CRS and asthma (Tieu et al., 2009; Lambrecht and Hammad, 2012). The presence of bacterial and fungal biofilms increased mucosal IgE in CRS patients, suggesting that these biofilms interact with the host immune system and perpetuate chronic inflammation (Foreman et al., 2012).

CRS is phenotypically classified into two groups based on the presence or absence of nasal polyps. This clinical classification of CRS patients was initially reflected immunologically with a predominance of T_H_2 cells and eosinophils in CRS patients with polyps (CRSwNP) and a predominance of T_H_1 cells and neutrophils in CRS patients without polyps (CRSsNP). However, recent studies using molecular methods have demonstrated up to 10 distinct endotypes of patients using markers of T_H_1, T_H_2, T_H_17, eosinophil, and neutrophil activation (Tomassen et al., 2016). A separate study found four distinct groups of CRS patients characterized by distinct bacterial communities, each conferring a unique immune response (Cope et al., 2017). *Corynebacteriaceae*-dominated communities conferred increased *IL-5* gene expression and a higher risk for nasal polyps. While these findings are helpful, it is also important to understand how the sinus microbiota drive or exacerbate observed inflammatory endotypes so new therapeutics can target the initiation of disease.

### Unified airway hypothesis

CRS often exists in the setting of concomitant lower airway disease, including asthma, and is frequently preceded by rhinitis. Upper respiratory infections can exacerbate asthma symptoms; bronchial hyperresponsiveness often occurs along with rhinitis (Leynaert et al., 2000; Passalacqua et al., 2001). Between 20 and 60% of CRS patients with nasal polyps have asthma (Larsen, 1996; Klossek et al., 2005). These observations have led to the “unified airway” hypothesis, which treats the respiratory tract as one organ rather than distinct organs affected by specific diseases. Supporting this hypothesis, medical and surgical treatment in the upper airways for CRS frequently results in reduced asthma symptoms. Therefore, respiratory inflammatory disease can be considered a disorder of the entire respiratory tract. Dysbiotic (altered) microbial communities have been described in the upper airways of CRS patients and in the lower airways of asthmatic patients, however, the microbiota of these two sites have not yet been examined in parallel. Below, we will discuss recent findings in CRS and asthmatic microbiota and potential interactions between these important microbial populations.

## Airway bacterial microbiome

The upper and lower airways harbor niche-specific microbial communities that relate to health status (Lemon et al., 2010; Huang and Boushey, 2014). Recent studies of the sinonasal microbiome generally demonstrate a loss of bacterial diversity and concomitant enrichment of pathobionts, resident microbes with pathogenic potential, in the sinuses of patients with CRS (Biswas et al., 2015; Cope et al., 2017; Lal et al., 2017; Wagner Mackenzie et al., 2017). Abreu and colleagues found that the sinus microbiome was characterized by reduced microbial richness and depletion of taxa associated with healthy individuals, such as lactic acid bacteria (Abreu et al., 2012). This depleted CRS microbiome was enriched with the pathobiont *Corynebacterium tuberculostearicum*. In a murine model, *C. tuberculostearicum* increased host mucin secretion, potentially increasing factors such as nutrient availability and adherence. Larger studies of upper airway microbiota in CRS patients demonstrate a heterogeneous, compositionally distinct microbiota which relate to distinct host immune responses (Cope et al., 2017; Lal et al., 2017). Studies comparing the lung microbiome of asthmatics and healthy controls found increased bacterial burden and diversity among asthmatics and a compositional shift in the bacterial microbiome composition characterized by enriched *Proteobacteria* (Huang et al., 2011; Han et al., 2012; Durack et al., 2017). Polymicrobial biofilms consisting of bacteria and fungi have been observed on sinonasal mucosa using fluorescence *in situ* hybridization, demonstrating that these taxa exist in mixed-species mucosal communities (Sanderson et al., 2006; Doble et al., 2007). More research is required on the upper and lower airway microbiota, and, in particular, on the relationship between sinonasal and pulmonary microbiota and mycobiota. The function and composition of these communities may shed light on the heterogeneity observed in CRS.

## Airway fungal microbiome

While the bacterial communities in airway inflammatory disease have been more extensively studied, fungal microbiota are still poorly characterized. This is due, in part, to the challenges faced by researchers who study the airway mycobiome. Low fungal biomass in the airways and databases that are curated from GI or environmental sources affect sample collection, DNA extraction, library preparation, and sequence analysis. In other body sites such as the GI tract, fungal dysbiosis has been implicated in diseases including inflammatory bowel disease. Although outside the scope of this perspective, gut mycobiome dysbiosis and interaction with the host immune response has been extensively reviewed (Underhill and Iliev, 2014; Mukherjee et al., 2015). In the airways, similarities between allergic fungal rhinosinusitis and ABPA illustrate the ability of fungi to act as antigens and invade the respiratory tract.

Studies on the fungal microbiome in CRS and asthma suggest that fungal communities in the airways likely influence host health. *Malassezia*, a known pathobiont on skin (Findley et al., 2013), is the predominant fungal genus in the sinonasal cavity in healthy individuals, CRS patients, and patients with allergic rhinitis (Cleland et al., 2014; Jung et al., 2015; Gelber et al., 2016). Lower abundance fungi in the sinuses of CRS patients include *Aspergillus, Alternaria, Fusarium*, and *Saccharomyces* (Cleland et al., 2014). A recent study using ITS gene sequencing found fungal presence in the sinuses of 63% of CRS patients and the predominant fungal genus in this study was *Aspergillus* (Zhao et al., 2017). PCR evaluations of *Aspergillus*, however, have demonstrated the presence of this fungus only in the sinuses of CRS patients with a known fungal subtype of CRS (Gelber et al., 2016). These discrepancies may be due to challenges related to fungal databases for human-associated ITS sequences. Analysis of the nasal vestibule of patients with AR and healthy individuals demonstrated increased diversity of fungal communities in AR characterized by preponderance of *Malassezia* (91–99% of 18S rRNA gene sequences) with lower abundance *Aspergillus* and *Alternaria* (Jung et al., 2015). In asthma, the lung mycobiome composition is altered between patients with severe asthma, ABPA, asthma with fungal sensitization (SAFS), and mild asthmatics. In ABPA, *Pseudomonas* abundance and fungal diversity both increased. Severe asthmatics were characterized by enrichment of *Aspergillus;* the relative abundance of *Aspergillus* increased approximately 15-fold compared to mild asthmatics (Chishimba et al., 2015). These results suggest that bacterial and fungal communities change in parallel in subsets of human disease. Future studies examining paired upper and lower airway microbiota (bacterial and fungal) in individuals with CRS and asthma are warranted.

*Aspergillus fumigatus* is often involved in the development of allergic fungal rhinosinusitis (AFRS) specific subtype CRS that accounts for an estimated 6–9% of all CRS patients undergoing surgery (Schubert, 2004). Patients with AFRS present with hyperplastic sinus disease, characterized by chronic eosinophilic-lymphocytic inflammation and nasal polyps. In AFRS, fungal hyphae are found within allergic mucin. Sinus cultures obtained in surgical patients reveal the presence of fungal taxa such as *Bipolaris spicifera, Alternaria*, and *Aspergillus*. AFRS bears many similarities to ABPA in its immunopathology, treatment, and outcomes. ABPA and AFS patients have elevated levels of serum IgE, and ABPA patients have elevated *A. fumigatus*-specific IgE and IgG. Allergic mucin in AFRS patients is histologically identical to the bronchial mucus plugs in ABPA patients (Schubert, 2004). In ABPA, immunological hypersensitivity is higher relative to AFS, possibly due to differences in the diseased organ or etiological fungus.

Concurrent upper and lower airway disease may be a singular inflammatory process caused or mediated by microbial communities. Microbes or their metabolites might translocate between the upper and lower airways. Interactions between microbes, the environment, and the host inflammatory response could alter the mucus-associated microbial communities, causing dysbiosis. Fungi involved in these allergic fungal disorders, along with associated bacteria such as *S. aureus*, viruses, or other microbes, may act as the source of microbial T-cell superantigens. Fungal contributions to chronic inflammatory airway diseases are an emerging research interest, one which may advance the understanding of such unified airway disorders and treatment for patients with allergic airway disease.

## Fungal-host and fungal-bacterial interactions and host health

Bacteria and fungi co-inhabit the human body and mounting evidence suggests that microbial interactions in a given niche can influence human health and disease. Bacterial co-colonizers can directly affect fungal morphology (Peters et al., 2012), survival (Romano and Kolter, 2005; Harriott and Noverr, 2009), growth (Kerr et al., 1999), virulence (Schlecht et al., 2015), and attachment to host epithelia or other microbes (El-Azizi et al., 2004; Morales and Hogan, 2010). Current investigations have focused on bacterial interactions with the opportunistic pathogen *Candida albicans*. In the oral cavity, *C. albicans* selectively attaches to the bacterium *Streptococcus gordonii* to increase attachment and growth of the fungi (Holmes et al., 1996). *In vitro* experimental studies have demonstrated that *S. aureus* and *P. aeruginosa* selectively attach to *C. albicans* hyphae but not yeast (Peters et al., 2012; Schlecht et al., 2015), and these interactions have distinct outcomes. *S. aureus* attachment to *Candida* hyphae results in increased invasion of tissue (Schlecht et al., 2015), while *P. aeruginosa* forms biofilms on the hyphal cells, killing the fungus (Hogan and Kolter, 2002). Physical sensing and Quorum sensing (QS) through small molecules that allow microbes to respond to environmental stimuli play important roles in mediating these interactions. For example, the *C. albicans* QS molecule farnesol inhibits *Pseudomonas* quinolone signal (PQS) and virulence factor production (Cugini et al., 2010). *C. albicans* can respond to *P. aeruginosa* QS molecules, specifically 3-3-oxo-C12 homoserine lactone, which prevents hyphal formation in the fungus (Hogan et al., 2004). These species-specific interactions through physical contact and QS molecules likely affect microbial virulence or biofilm formation in host upper or lower airways. Ongoing research in our lab seeks to explore these interactions.

We are interested in interactions between sinonasal-associated fungi and bacterial co-colonizers, including *Malassezia* sp. (Mowat et al., 2010; Jung et al., 2015; Gelber et al., 2016). *Malassezia*, a dimorphic yeast implicated in a variety of conditions including dandruff and atopic dermatitis, is present in the upper airways (Cleland et al., 2014; Gelber et al., 2016). *Malassezia* species are known to be immunomodulatory and encode at least 13 different allergens; these fungi can exist as commensals and can be immunosuppressive (Ashbee and Evans, 2002) or as pathobionts and can become immunostimulatory (Gaitanis et al., 2012). Similar to CRS, *Malassezia dermatis* often co-occurs with *S. aureus* in atopic dermatitis, perhaps interacting with bacteria in driving skin inflammation. Further research is required to identify these *Malassezia* at the species or strain level and to compare populations between healthy subjects and those with allergic airway disease. Mechanistic studies are needed to reveal the interactions between *Malassezia*, bacterial community members, and the host immune system.

Airway epithelial cells and phagocytes express pattern recognition receptors, including C-type lectins, that sense and respond to components of the fungal cell wall. Dectin-1, a pattern recognition receptor expressed by dendritic cells, neutrophils, and macrophages, recognizes the fungal polysaccharide β-1,3 glucan motif found on fungal cell walls. Dectin-1 may mediate host immune responses to these fungi. In mice, loss of dectin-1 resulted in more severe colitis compared to wild-type mice. DSS-induced colitis was associated with increased *Candida* and *Trichosporon* and decreased *Saccharomyces*. Fungi were also found to invade inflamed tissues in dectin-1 KO mice, but not in wild-type mice. When given the antifungal fluconazole, dectin-1 KO mice had milder symptoms (Iliev et al., 2012). These results indicate that fungi contribute to the aggravation of inflammatory responses in colitis, and dectin-1 is an immune mechanism that regulates fungal community composition. Another C-type lectin that specifically recognizes *Malassezia* has recently been described (Yamasaki et al., 2009). When expressed on macrophages, Mincle (also called Clec4e and Clecsf9) sensing of *Malassezia* induces pro-inflammatory responses (Yamasaki et al., 2009), although this receptor has also been implicated in T_H_2-polarization upon activation (Geijtenbeek and Gringhuis, 2016). Mincle-deficient mice showed a 2-4 fold reduction of IL-10, TNF-alpha, and MIP1 when challenged with *Malassezia* but not *A. fumigatus* (Yamasaki et al., 2009) Host-associated commensal fungi, therefore, specifically interact with the mucosal immune system, maintaining host and microbial homeostasis.

Fungal microbial communities in the GI tract can also affect the development of respiratory disease. Mice treated with antifungals exhibited increased development of allergic airway disease along with increased disease severity in models of colitis (Wheeler et al., 2016). The microbiomes of these mice revealed a restructuring of fungal and bacterial communities characterized by decreased *Candida* (2.25-fold) and increased *Aspergillus* (2.5-fold), *Wallemia* (6-fold), and *Epicoccum* (4-fold). Oral supplements of *A. amstelodami, W. sebi*, and *E. nigrum* recapitulated the effects of antifungal drugs to exacerbate allergic airway disease as measured by increased pulmonary eosinophils and lymphocytes and elevated serum IgE (Wheeler et al., 2016). Colonization of the gut by *Candida albicans* has also previously been shown to influence allergic airway disease and asthma in a mouse model, perhaps through secretion of prostaglandin-like immunomodulatory molecules (Noverr et al., 2005; Huffnagle, 2010; Marsland and Salami, 2015). Thus, fungal dysbiosis in the gut can affect disease development and exacerbation in the respiratory tract, indicating that a healthy mycobiome mediates immune responses throughout the body. Whether these effects reflect a direct action of fungi on the host or occur indirectly by altering the bacterial microbiome composition is unknown. Further studies are needed to elucidate the mechanisms for these responses and to determine whether fungal communities in other organs can influence allergic airway disease.

## Future directions

The evidence for the influence of dysbiotic fungal and bacterial microbiota interactions to drive or exacerbate chronic airway inflammatory disease is compelling. These findings open up the potential for targeted manipulation of the airway or gastrointestinal tract microbiota to improve airway health and manage airway disease in patients with CRS, asthma, and cystic fibrosis, among others. Current mycobiome studies focus on comparisons in healthy vs. diseased models, but more research needs to occur to understand the interactions between fungal and bacterial communities with the host immune system in host body sites. Fungi produce diverse secondary metabolites that can affect bacteria, while bacteria can keep the mycobiome in check by producing substances that inhibit the yeast to hyphae transition of fungal pathobionts such as in *C. albicans*.

**Figure 1:**
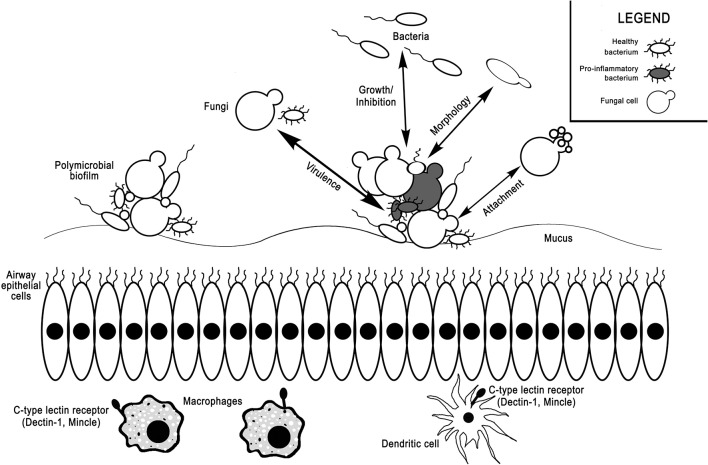
Interactions between the bacterial microbiome, mycobiome, and host immune system in the airways. Bacteria and fungi coexist in mucosa-attached polymicrobial biofilms in the airways. Fungi can selectively induce or inhibit growth of various bacterial taxa, increase bacterial virulence factors, alter bacterial morphology, or act as attachment sites for bacteria. Vice versa, bacteria can also alter fungal growth, virulence, morphology, and attachment. C-type lectin receptors on macrophages and dendritic cells, such as dectin-1 and Mincle, can sense fungi and mediate host inflammatory responses.

Additional research is needed to define the functional effects of the mycobiome (Figure 1). Rather than focusing on taxonomic diversity, studies comparing microbial community function are potentially more useful. Further understanding of the contribution of the mycobiome and bacterial-fungal interactions should move beyond the GI tract into respiratory tract, where fungal dysbiosis has been shown to influence disease development and exacerbate symptoms. A variety of methods, combining metagenomics and sequencing approaches, experimental models, and functional studies should be used to clarify mechanisms by which the mycobiome impacts health and disease. In particular, focusing on microbial interactions with mucosal surfaces and their influence on local immune response likely will provide additional insights into the role of microbes in inflammatory disease of the upper and lower airways. Because fungal dysbiosis can have as great an effect on the host as bacterial dysbiosis, we cannot overlook the contributions of these largely unexplored fungal communities in our search to understand the microbial mechanisms behind health and disease.

## Author contributions

IZ drafted and revised the manuscript. EC conceived, wrote, and revised the manuscript. BB conceived, wrote and revised the manuscript. AG conceived and revised the manuscript. SP conceived, wrote, and revised the manuscript.

### Conflict of interest statement

The authors declare that the research was conducted in the absence of any commercial or financial relationships that could be construed as a potential conflict of interest.
